# Parent priorities for research and communication concerning childhood outcomes following preterm birth

**DOI:** 10.12688/wellcomeopenres.16863.2

**Published:** 2021-09-30

**Authors:** Lorna Ginnell, James P. Boardman, Rebecca M. Reynolds, Sue Fletcher-Watson

**Affiliations:** 1 Salvesen Mindroom Research Centre, University of Edinburgh, Edinburgh, UK; 2 MRC Centre for Reproductive Health, University of Edinburgh, Edinburgh, UK; 3 Centre for Cardiovascular Science, University of Edinburgh, Edinburgh, UK

**Keywords:** Preterm birth, Childhood outcomes, Parent priorities, Research, Communication

## Abstract

**Background:** Children born preterm (before 37 weeks of gestation) are at risk for several adverse childhood outcomes. Parent priorities for research into these outcomes, and preferences for receiving information about these risks, have not previously been established. Here we report the results of an online survey designed to understand parent priorities for research and their preferences for receiving information about childhood outcomes.

**Methods:** An online survey was circulated through social media and was completed by 148 parents of preterm children between the ages of 0 and 12 years from around the United Kingdom (UK). Survey questions were in the form of rating scale, multiple choice, ranking or open-ended free text questions. Descriptive analysis was applied to the quantitative data. Illustrative quotes were extracted from the qualitative free text data and a subset of these questions were analysed using framework analysis.

**Results:** Parent priorities for research centre around identification of factors which can protect against or improve adverse cognitive or developmental outcomes. The majority of parents would prefer for communication to begin within the first year of the child’s life. Parents reported a knowledge gap among health visitors, early years educators and schools.

**Conclusions:** In order to align with parent preferences, research should prioritise identification of protective factors and the development of effective interventions to improve outcomes. Training for health visitors and educational professionals could improve the experiences of families and children.

## Introduction

Children who are born preterm are at risk for a number of neurodevelopmental, cognitive, educational and psychiatric difficulties during childhood and later in life ( Aarnoudse-Moens *et al*., 2009; Johnson & Marlow, 2011; Johnson & Marlow, 2017; Johnson *et al*., 2009; Johnson *et al*., 2011; Marlow, 2004). Research aiming to understand the manifestation of, and pathways to, these difficulties is essential for improving care and developing interventions. As such, families of preterm children are often asked to participate in research studies which can sometimes be accompanied by burdens such as the time commitment necessary to complete paperwork or attend appointments. 

Parent priorities and experiences may differ from those of clinicians and researchers, and so it is important to involve them in the research process. A meta-analysis concluded that parent opinions about which neonatal outcomes research should focus on differ from those of doctors and researchers ( Webbe *et al*., 2018). Some studies have taken parent input into consideration, but the majority of recent studies of this kind focus on the neonatal period ( Harvey *et al*., 2017; Webbe *et al*., 2018; Webbe *et al*., 2020). Much less is known about parent perspectives on preterm birth research across childhood. However, one recent study has focused on parent and other stakeholder priorities for childhood outcomes ( Zeitlin *et al*., 2020). Through a Delphi process, Zeiltin and colleagues found that themes relating to the socioemotional needs of children and parents were most highly ranked across all stakeholder groups. Parents ranked emotional well-being, social inclusion and education most highly while health care professionals prioritised care and outcomes following extremely preterm birth and the impact of impairments on quality of life and education. This finding reiterates the importance of capturing parent perspectives, since their priorities, attitudes and experiences may differ from those of clinicians and clinical academics.

There are a range of parental opinions which could and should be captured to inform research and practice in prematurity. Along with the outcomes being measured, longitudinal study designs and research with older children introduce a number of considerations that may influence parents’ decision to participate in research during childhood. For example, the different measures used, commitment required and children themselves becoming aware and expressing opinions about participation. Understanding parental motivators for participation in preterm research could be useful in tailoring recruitment and informed consent processes.

Finding out how parents of preterm-born children feel about research across childhood is important in and of itself, to facilitate the design of studies that are aligned with family priorities. However, this question interfaces with another – how much do parents know about long-term outcomes following preterm birth? Rates of diagnoses such as autism ( Limperopoulos *et al*., 2008) and attention deficit hyperactivity disorder ( Johnson *et al*., 2016; Pedro Franz *et al*., 2018) as well as sub-clinical dimensional problems with attention ( Anderson *et al*., 2011), and social cognition ( Zmyj *et al*., 2017) are increased in preterm-born children. Although risk for these and other problems is increased in this population, there remains a great degree of heterogeneity in outcome, with some children experiencing no known issues relating to their prematurity. Uncertainty remains about how and when practitioners should share information with parents about risks of this nature, especially in the absence of reliable early-life markers for later outcome. Parents of preterm children experience higher rates of parenting stress, anxiety and depression, even several years after the birth ( Treyvaud *et al*., 2014). The emotional impact of this type of information on the family must be carefully considered and best practices should be centred around the views and preferences of families. Parents in a European cohort, including participants from the UK, have expressed dissatisfaction with follow-up care and suggest that better communication with parents could improve this ( Seppänen *et al*., 2021). These considerations are relevant to researchers as well as health care professionals. Harvey *et al*. (2017) found that parents reported positive experiences of participation in neonatal research, but that there is a need to balance information provision with the emotional needs of the parent. Understanding the preferred timing and format of delivery of sensitive information could improve family experiences of clinical care and of research participation.

The current study aims to i) survey the attitudes and preferences of parents of children who were born preterm towards taking part in research and ii) understand preferences surrounding communication of information about long-term outcomes.

## Methods

### Participants

Respondents were a UK-based sample of 148 parents of preterm children (born before 37 weeks of gestation, according to parent report). Respondents were excluded if they lived outside the UK, if their child was born after 37 weeks of gestation or was over the age of 12 years at the time of survey completion.

### Materials

The survey collected data on parent and child demographic information, child difficulties, and parent preferences, opinions and experiences relating to research participation and communication about long-term outcomes. The survey structure was informed by a similar survey designed to capture attitudes of the autism community (including parents of autistic children) towards research into early autism ( Fletcher-Watson *et al*., 2017). The content of the survey was informed by the experience of the authors including that of data collection with children born preterm, informal discussions with parents around the topics explored in the survey, and drew on the outcome measures of a contemporary UK-based longitudinal cohort study of preterm birth ( Boardman *et al*., 2020).

The survey consisted of four broad sections. Section one collected demographic information about the respondent e.g., gender and age leaving full-time education. Section two focused on child demographic information e.g., gender, age, gestational age at birth. Section three consisted of three ranking style questions about preferences and opinions surrounding research, and one open ended question about past research experiences. For ranking style questions, participants were presented with a number of response options and asked to rank the options in order of importance. Section four consisted of multiple choice and rating scale questions about child cognitive and behavioural profile, including diagnostic status. This section also included two ranking questions about when and how parents should be communicated with about long-term outcomes, and finally two open ended questions giving respondents the opportunity to share any additional information or comments about their child or the survey. The survey was checked for comprehensibility by a parent who was external to the research team and academia resulting in the addition of further instructions on how to answer ranking style questions and correction of minor typos. No further piloting was carried out. A PDF of the survey is available as extended data ( Ginnell, 2021).

### Procedure

Ethical approval was granted by the University of Edinburgh School of Philosophy, Psychology and Language Sciences Research Ethics Committee [300-1920/2]. An integrated information sheet and consent form were presented to participants at the beginning of the survey and consent was mandatory for progression through the survey. The survey was hosted on the web-based platform Online Surveys and was distributed via Facebook and Twitter, including private groups or public accounts of UK based charities and parent support groups and through the professional networks of the authors. The survey was open between June and November 2020. It took approximately 25 minutes to complete.

### Analysis

Data were downloaded in excel format and analysed using R version 4.0.1. Data from quantitative ranking style questions are represented visually as mean ranking value per response item. Multiple choice question data are represented as the frequency of respondents selecting each choice. Rating scale data are represented as the percentage of respondents that selected each rating option. Quotes from open text format questions were used illustratively to support and interpret quantitative findings.

Some open text format questions were analysed using Framework Analysis. Framework analysis is a five-step process for organising and summarising patterns in qualitative data ( Ritchie & Spencer, 1994). The output is a set of themes which are driven in part by predefined research questions but can also incorporate unanticipated patterns in the data. First the researcher (LG) familiarised themselves with the data, noting themes or impressions. A set of themes were decided upon based on both the research questions and on patterns identified in the data during familiarisation. Codes were assigned to individual quotes to indicate which theme they represented, and data were organised based on codes and associations. Key characteristics of the data were identified, and interpretations and explanations were proposed. Microsoft Excel and Miro were used for the qualitative analysis.

***CHallenge score*.** In order to explore whether responses differed for respondents whose child does or doesn’t experience challenges related to their prematurity, a “challenge score” was derived to quantify the degree of difficulty experienced by individual children. Higher scores indicated more frequent and more serious difficulties.

Respondents were asked whether their child experiences challenges in 11 different areas (motor skills, memory, attention, learning, language, emotions, behaviour, socialising, stress, anxiety, depression). Challenges were defined for parents as things a child can’t do or were delayed in doing relative to other children their age (e.g., language delay) or things they have more trouble with compared to other children (e.g., behavioural issues). Response options included not at all, a little, a lot or not applicable. A numeric value of 2 was assigned to “a little” answers and a value of 3 was assigned to “a lot” answers. The challenge score was calculated by summing all “a little” and “a lot” answers across the 11 domains for each participant. A minimum score of 0 indicates no challenges in any of the areas specified and a maximum score of 33 indicates “a lot of trouble” in all listed challenge areas. A higher score indicates more numerous and / or more impactful challenges but does not differentiate between number and degree of difficulties. Respondents were categorised based on their challenge score, with a score of 10 or above qualifying for inclusion in the high challenge group. Children younger than 2 years of age were excluded from this categorisation as challenges in the majority of the domains asked about would not be apparent at this age. Responses to ranking questions were visualised by group in order to assess whether rankings differed between low and high challenge groups.

## Results

### Participant characteristics

Of 159 initial respondents six were excluded due to the child being older than 12 years and five were excluded because the child was born outside of the UK. Of the remaining 148 UK respondents, the number of respondents from England, Scotland, Wales, and Northern Ireland respectively were 58, 49, 4 and 1. 38 participants did not specify which UK country their child was born in. Demographic characteristics of the final sample are summarised in Table 1 ( Ginnell, 2021). 

**Table 1.** Demographic characteristics of the sample. SD=standard deviation; M:F=male:female ratio.

Characteristic	n=148
Mean gestational age at birth/weeks (SD, range)	30.37 (3.93, 22-36)
Mean child age/years (SD, range)	4.76 (2.84, 0-12)
Child gender M:F	96:52
Respondent gender M:F	6:142
Mean respondent age leaving full-time education/years (SD, range)	20.88 (2.95, 16-33)

### Parent preferences and experiences concerning research

When asked about previous experiences participating in research, the majority of respondents reported having positive experiences. Families appreciated when studies were well explained and unobtrusive: e.g., *“well explained and were of no inconvenience to us as parents and not at all invasive or challenging to our daughter”* [R130]. Less positive comments related to sharing of study findings, with several respondents expressing disappointment or frustration that results were not shared with them “ *disappointed that we weren’t contacted with findings of the research study” [R39]*.

**Figure 1.**
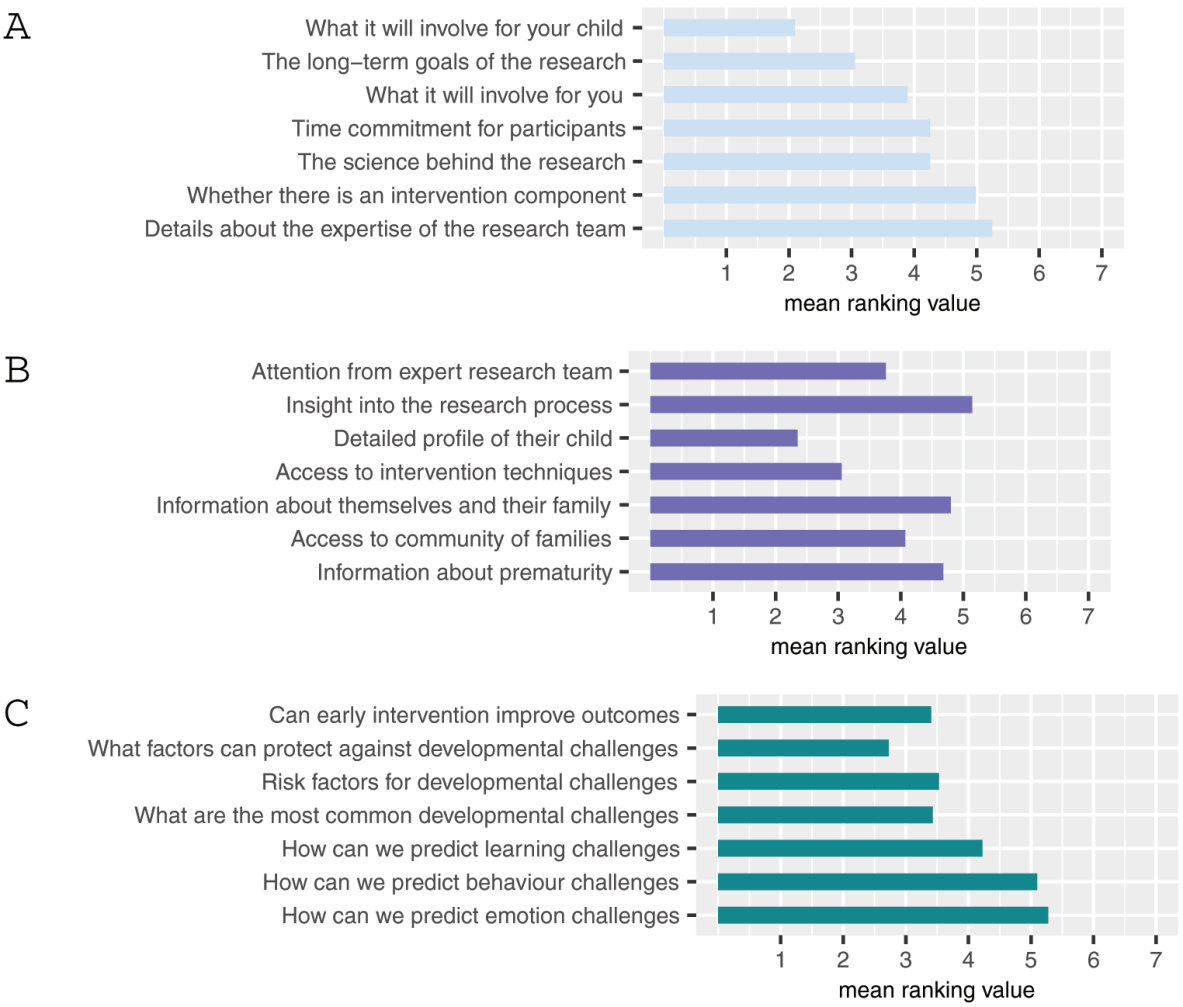
Preferences and opinions surrounding research. ( **A**) What information would be most important when making your decision about whether to become involved or not? ( **B**) What direct benefits should people who take part in research studies receive? ( **C**) What are the most important questions scientists should be asking about prematurity as children grow up? For each question ( **A**, **B**, **C**) participants were asked to rank the available response options in order of importance. Lower scores indicate higher importance. Values are mean ranking, listed in order of their modal ranking.

Respondents’ main considerations when deciding whether to take part in research are what it will involve for their child but also the long-term goals of the research ( Figure 1A). Though there was notable variability in these data suggesting less agreement between respondents. Nevertheless, this demonstrates that parents are motivated to participate for altruistic reasons rather than personal benefit. Some respondents expressed a feeling of reward at having contributed toward improving care for families in the future “ *It was emotional but very rewarding knowing the research could help others”* [R126].

However, when asked specifically about direct benefits for participants the majority of respondents ranked “a detailed profile of their child” as their highest priority ( Figure 1B).

Finally, parents prioritised research questions which aim to identify protective factors rather than risk ( Figure 1C).

### Communication preferences

**Figure 2.**
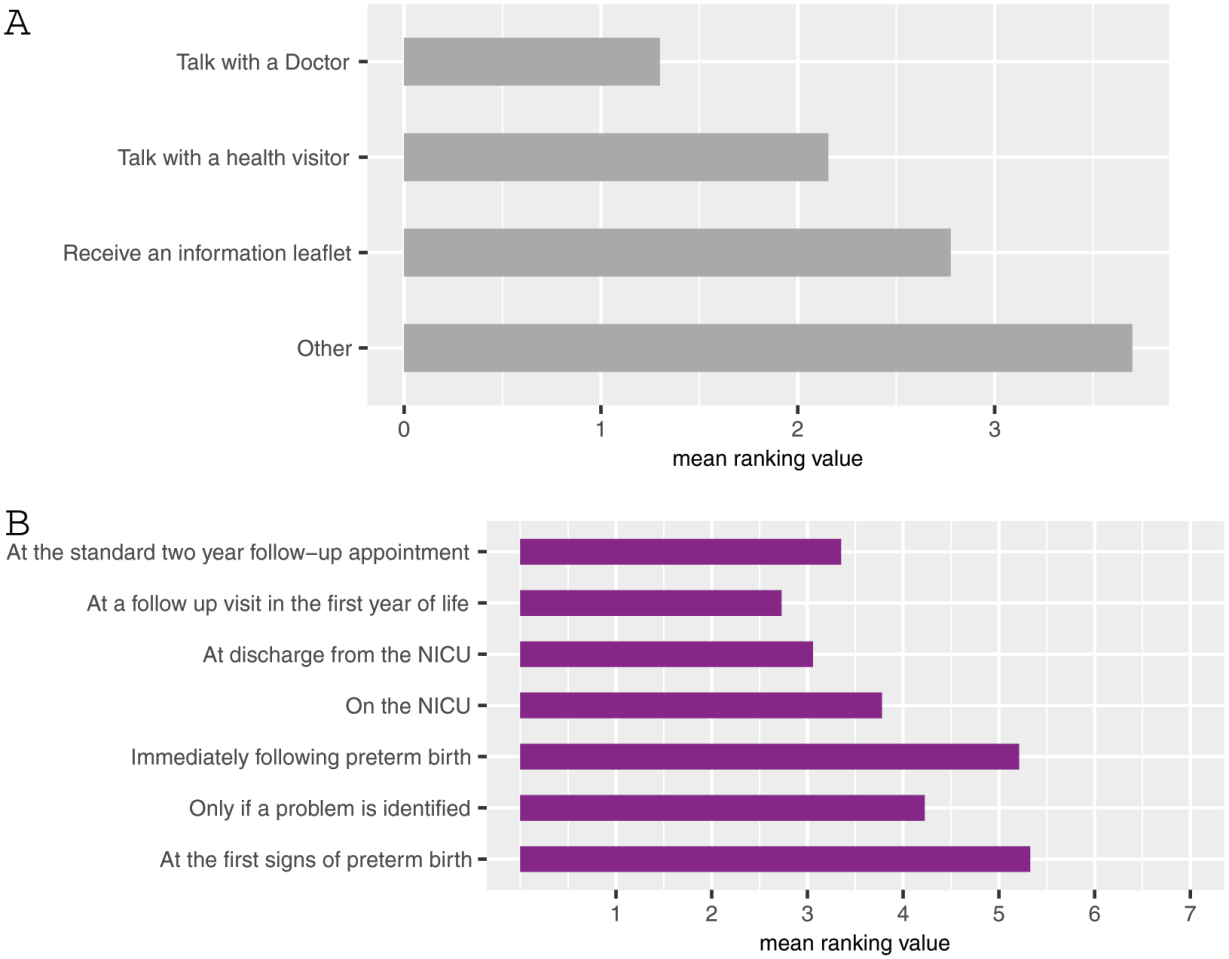
Communication preferences. ( **A**) What would be the best way to share information about [long term challenges] with parents? ( **B**) When would be the best time to tell parents that these problems [long-term challenges] are more common in preterm children? For each question ( **A**, **B**) participants were asked to rank the available response options in order of importance. Lower scores indicate higher importance. Values are mean ranking, listed in order of their modal ranking.

When asked about when and how they would like to be communicated with about the long-term challenges that are sometimes associated with preterm birth, respondents rated speaking to a doctor ( Figure 2A) within the first year of the child’s life ( Figure 2B) most highly.

Responses to the open-ended questions revealed important additional insights into parent experiences and opinions around communication of challenges. While some felt that *“parents should be much better informed“* [R39] or that they would have benefitted from more information: *“being spoken to by a medical professional about the possible risks for all the above areas would have been better for me”* [R24], some felt they were appropriately informed *“[doctors] were honest and open about our daughter potentially having difficulties as she developed from the day she was born and this really helped us come to terms with it”* [R95]. 

Some respondents felt that too much focus was placed on their child’s challenges and called for an emphasis on the potential for intervention and positive progress *“it's important to give balanced information, and be careful around language used. Words have a long-lasting impact and can cause parents unnecessary worry. Concentrate on the positive of early intervention to support a child”* [R59]. Similarly, some respondents expressed concerns about the unnecessary worry that can result from highlighting problems that may never arise for their child *“speculation as to what might be can be unhelpful in upsetting parents”* [R20].

Others felt that too much information provided too early on could be overwhelming *“On the NICU we were just focussed on him staying alive another day, his long-term outcome wasn’t as important until he was much older”* [R128]. One respondent suggested that ongoing discussions with parents could help to avoid this *“Discussion with parents needs to be ongoing, not information delivered at a single point. Information delivered throughout neonatal journey (which includes beyond NICU) will capture the needs of all parents and enable families to take onboard information at a time ready for them”* [R33]. There is clearly a delicate balance to be achieved in order to provide parents with information in a way that is sensitive and appropriately timed.

### Challenges

**Figure 3.**
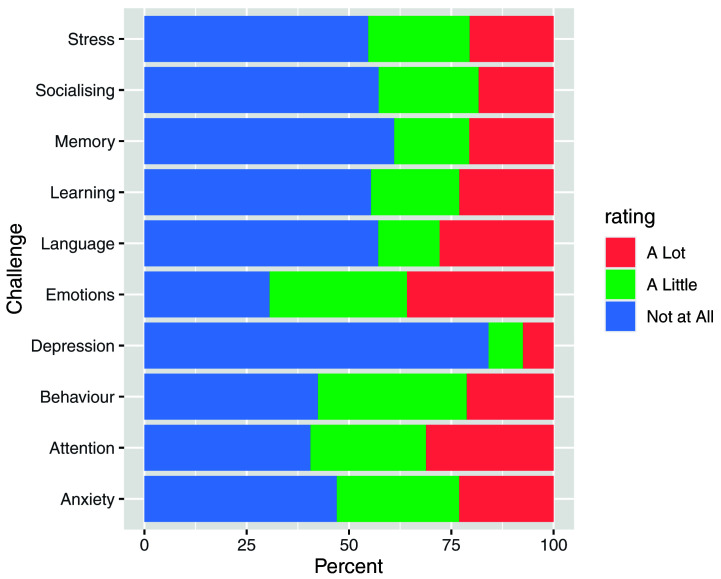
Answers to the question: Relative to other children their age, have you noticed your child having trouble with any of the following things?

**Figure 4.**
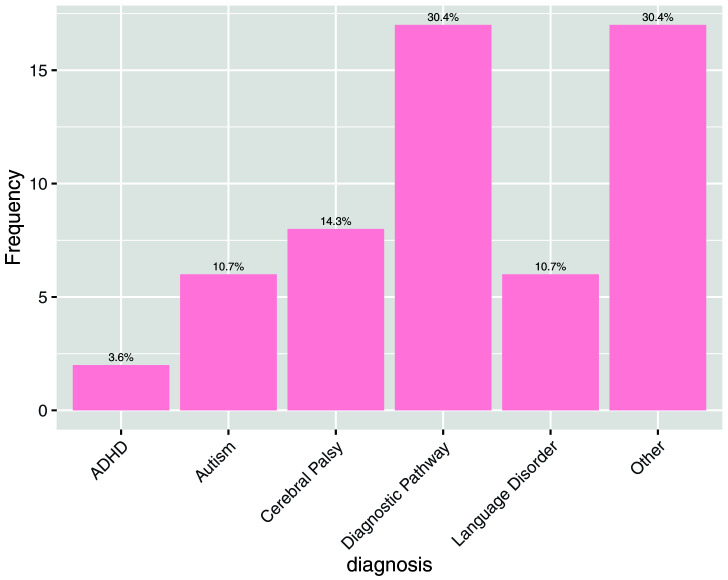
Answers to the question: Does your child have a diagnosis? “Other” included Developmental Coordination Disorder, Downs Syndrome, Dyslexia, Dyspraxia, Global Developmental Delay, Hemiplegia and Sensory Processing Disorder.

**Figure 5.**
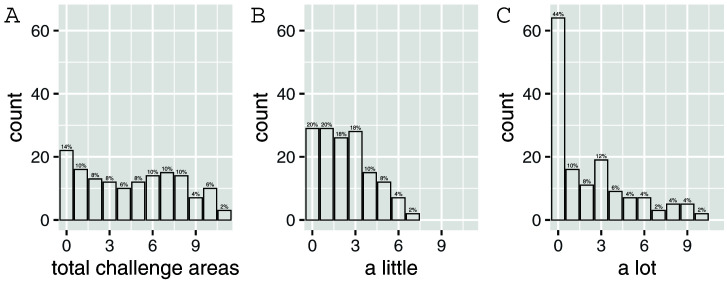
The number of participants reporting challenges in multiple areas. ( **A**) Number of participants reporting “a little” or a “lot” of trouble in 0-11 challenge areas. ( **B**) Number of participants reporting “a little” of trouble in 0-11 challenge areas. ( **C**) Number of participants reporting “a lot” of trouble in 0-11 challenge areas.

In order to further understand the characteristics of this sample, respondents were asked whether their child experienced challenges in a number of areas ( Figure 3) or if they had any diagnoses ( Figure 4). Figure 5 shows the number of participants reporting challenges in multiple areas.

A challenge score was calculated for each participant and participants were grouped into high and low challenge score categories. In order to understand the influence of challenge category on opinions about research and communication, modal ranking values for each response item were visually compared between groups. A difference in modal value of two points or more between challenge groups was considered large enough to warrant statistical investigation. The modal value for the top two priority items did not differ between groups by more than one point for any item and so no statistical tests were justified. This indicates that priorities and opinions about research and communication do not differ between parents whose children experience varying degrees of difficulties relating to their prematurity.

### Knowledge and support

Respondents were given the opportunity to share any additional thoughts before completing the survey. Two main themes arose from the responses: 1. a knowledge gap amongst some health care and education professionals, 2. experiences of concerns being dismissed leading to difficulty accessing support.

Some parents expressed a need for greater education and training for health visitors *“in our experience and most people I've talked with, health visitors have a very big skills gap in understanding about premature babies”* [R12], early years education *“I would like to know if there is any information that can be shared with early years as I feel not all practitioners take this into account when working with a child”* [R54] and schools *“I think a key aim would be to increase knowledge in primary schools particularly SENs [special educational needs] teachers*” [R12]. Unfortunately, this lack of knowledge has led to some parents feeling that their concerns have been dismissed and that their chid had missed out on support as a result *“Very difficult to find anyone who understands longer term consequences of prem birth. My experience has been complete disregard of prematurity when discussing my children’s behavioural and emotional difficulties. Resulting in a complete lack of support and lots of stress within the family”* [R34]. 

Other respondents commented on the lack of routine follow-up as children grow up *“it would be great if there was a further support system in place for the future, whether it be something formal like annual review or just having somebody to be able to contact regarding concerns”* [R97].

## Discussion

This study adds important additional insights to the sparse literature regarding parent priorities for research into childhood outcomes following preterm birth, and preferences for how and when to be communicated with about potential adverse outcomes. Findings from 148 UK respondents indicate that parent priorities for research centre around identification of factors which can protect against or improve adverse cognitive or developmental outcomes. Parents want to be informed about potential long-term challenges within the first year of the child’s life and feel it is important to also receive information about support and the potential for positive progress. Preferences for research and communication did not differ based on the degree of difficulty experienced by the child. Parents reported a lack of knowledge about prematurity among certain professionals involved in their children’s care and resultant difficulties accessing support. 

### Implications for research

Our findings indicate that in order to align with parent preferences, research should focus on resilience rather than risk, and work towards the development of effective interventions. This is in line with the James Lind Alliance findings on parent priorities for neonatal research, where the top three priorities related to intervention and / or prevention ( Oliver *et al*., 2019). This intersects with findings of a desire for information provision relating to adverse outcomes to be balanced with information about opportunities for intervention and support. The nature of the research process means that the identification of risk is often a necessary prerequisite for intervention trials. Clear and sensitive communication with participants about these research pathways may improve their experience and willingness to participate as we also found that parents prioritise the long-term goals of a study when considering participation in research.

The finding that parents would like to receive individualised information about their child’s profile as a result of participating in research is perhaps linked to their reports of dissatisfaction with the support they can access through clinical or educational routes. Perhaps direct pathways for communication and referral between research and clinical services should be expanded. These pathways are already in place for referral of actionable clinical findings and incidental findings. Expansion of these pathways to include further information about a child’s profile would need to be carefully managed to protect confidentiality and data integrity. The purpose of this information sharing would need to be carefully considered, and only actionable facts communicated. Many of the measures used in research are not suited to this approach as they are often not diagnostic in nature and designed for interpretations at a group level. Regulations set out by research ethics committees limit what researchers are permitted to share. Clearer communication with research participants around these methodological and ethical constraints during the informed consent process could improve their experience of the research process.

In terms of direct communication with study participants, researchers have a responsibility to communicate group level findings to their participants but should perhaps also consider ways to share individual level data responsibly and securely. The constraints mentioned above around data sharing with clinical stakeholders also apply here, with the added important consideration of the impact on parents of receiving information that is not readily interpretable. Human and economic resources would need to be invested to ensure that information sharing is caried out in an accessible, clear and sensitive way. This would necessarily divert resource away from other research activities and so more work is needed to fully understand the costs and benefits of this approach.

As there are less stringent constraints placed around sharing of group level findings and given the knowledge gaps among early years education and schools highlighted by this survey, there is an opportunity for researchers to play a role in bridging these gaps by communicating their findings to other stakeholders in the preterm child’s journey. This would increase awareness of prematurity and may encourage greater engagement with other resources designed for these professionals, for example the PRISM resource: Preterm Birth Information for Educational Professionals.

### Implications for clinical and educational practice

Although findings indicate that the majority of parents would like to receive information about potential long-term challenges within the first year of their child’s life, conflicting opinions suggest that what is right for one family may not be best for another and some raised concerns around unnecessary worry caused by “ *speculation as to what might be*” [R20]. Allowing parents to choose when they engage with information of this nature, by offering follow-up at various stages in the child’s journey and having agility within follow-up services to allow information sharing at different times, would allow parents to take in information at a time that is right for them. This is in line with National Institute for Health Care Excellence (NICE) guidelines on an individualised approach to healthcare ( NICE, 2012) which are recommended in their guidelines for preterm follow-up ( NICE, 2017). This will not only reduce stress and improve families’ experience and care, but it will also increase parents’ ability to fully engage with the information. Standard written materials for parents to read at a time of their choosing, augmented by flexibility within follow-up services to allow for discussion of these materials could be one solution. Where possible, information about risk for adverse childhood outcomes should be carefully balanced with information about access to support and the potential for positive benefits of early intervention.

When given the opportunity to express any further thoughts or concerns, the themes of a knowledge gap amongst some health and educational professionals, and lack of access to support arose. Our expectation was that if parents are not informed about long-term outcomes through clinical services, then they may never learn of the consequences of preterm birth. In reality, parents are educating themselves and, in many cases, it is service providers who need to catch up with parent knowledge.

Health visitors acknowledge a lack of formal training and education about prematurity and how best to support parents with preterm babies ( Petty *et al*., 2019). As parents want to receive information about future challenges within the first year of their child’s life ( Figure 2 B), there is a tangible opportunity for integration of this information provision into existing pathways through upskilling of health visitors who, in terms of timing, are best placed to deliver it. Our respondents reported a preference for information provision to come from a doctor ( Figure 2 A), but perhaps if health visitors were more knowledgeable preferences would differ.

Similarly, a UK survey of over 500 educational professionals found that staff admit to a lack of knowledge about the long-term consequences of prematurity, with only 16% of those surveyed having received formal training and only 38% feeling sufficiently equipped to support a preterm child ( Johnson *et al*., 2015). Another study found that teachers’ confidence in supporting preterm children significantly increased after completion of an e-learning resource designed for educational professionals ( Johnson *et al*., 2019). Education and training for teaching staff would not only improve knowledge and awareness of prematurity and its consequences, but also increase points of contact for parents to voice concerns while improving care and support for individual children.

Increased understanding of prematurity may also help to address the experiences families reported around difficulty accessing support. Advanced knowledge of the outcomes associated with prematurity, along with information about individual students’ history of prematurity may prime teachers to pick up on subtle difficulties or increase their likelihood of acting on parent concerns. More work needs to be done to understand why existing pathways for communication and referral between different stakeholders do not seem to work for every child.

### Limitations & future directions

A potential limitation of this survey is the demographic profile of the respondents. Participants who have had past positive experiences of research are perhaps more likely to engage further with the research process than those who have had less positive research experiences, or none at all. In addition, the recruitment pathway may have encouraged participation from parents whose opinions may be reflective of specific experiences or demographics e.g., families who feel unsupported may follow charities such as those who shared the survey. Preterm birth is more common among families from lower socioeconomic brackets ( Bonet *et al*., 2013). The mean age of the respondents leaving full time education suggests that the majority of respondents were university educated and so may not be representative of the full social spectrum or the population most often affected by preterm birth. Though, despite a relatively high mean age, a broad range was captured ( Table 1). Furthermore, information about the race or ethnicity of the respondents was not collected. Future studies with more diverse recruitment pathways would be beneficial. A final demographic limitation is the relatively small sample size with particularly low representation of participants from Wales and Northern Ireland. Future larger studies should focus on increased representation from these geographical regions as well as capturing greater ethnic and socioeconomic diversity. 

The qualitative findings reported here are limited by the fact that the analysis was conducted independently by a single researcher. In order to minimise bias, future studies wishing to confirm these findings should involve more than one researcher. Qualitative findings suggest that parents hold contrasting opinions around some issues e.g., the timing of information sharing. Studies aiming to quantify the proportion of parents holding various views would be of interest.

A final limitation is the lack of a systematic process for selecting challenge categories, survey questions and response items. The specific challenges asked about will not represent the full range or perhaps the most salient of challenges faced by children born preterm. Respondents were given a finite number of responses to rank and although open ended questions provided the opportunity to share additional views, it is possible that parents may have other priorities and preferences that were not represented here. Future studies with a more flexible process for question and item selection that involves parents and other stakeholders would be beneficial. It would also be valuable to conduct similar studies in other parts of the world. If opinions are aligned, it would suggest that priorities for research and guidelines for communication and training could be coordinated on a larger scale. Surveys of this nature may be useful for longitudinal studies that wish to incorporate the preferences of their own participants specifically when designing next stages of their studies.

## Conclusions

In order to align with parent priorities, research should attempt to identify protective factors and interventions designed to improve childhood outcomes for children born preterm. More choice should be given to parents to decide when to engage with information about longer term adverse outcomes associated with prematurity. When information about risks is communicated to parents, it should be balanced with information about opportunities for support and intervention. Training for health visitors, early years educators and schools could improve the experiences of families and children.

## Data availability

### Underlying data

Qualitative data corresponding to open-ended free text format questions are not publicly available as they contain information that could compromise participant privacy and anonymity. These data can be requested from the corresponding author [LG, s1468169@ed.ac.uk] upon reasonable request.

Open Science Framework: Prematurity: Parent Engagement and Attitudes to Research (PEAR). https://doi.org/10.17605/OSF.IO/WJSK4 ( Ginnell, 2021).

This project contains the following underlying data:

- PEAR_data_anonymised.xlsx. (Quantitative data after the anonymisation process).

### Extended data

Open Science Framework: Prematurity Parent Engagement and Attitudes to Research (PEAR) Survey. https://doi.org/10.17605/OSF.IO/WJSK4 ( Ginnell, 2021).

This project contains the following underlying data:

- Parent priorities for research and communication concerning childhood outcomes following preterm birth.pdf
- PEAR_variable_key.xlsx
- PEAR data guide.pdf
- PEAR Survey.pdf
- Prematurity: Parent Engagement and Attitudes to Research (PEAR) Survey Report.pdf (an accessible report of the findings reported here)
- Problem Score.pdf

Data are available under the terms of the Creative Commons Zero "No rights reserved" data waiver (CC0 1.0 Public domain dedication).
