# Supplementary material for: Parent priorities for research and communication concerning childhood outcomes following preterm birth
Source: Wellcome Open Res. 2021 Sep 30;6:151. Originally published 2021 Jun 14. [Version 2] doi: 10.12688/wellcomeopenres.16863.2 (PMC8546737; doi:10.12688/wellcomeopenres.16863.2)
Supplement: Peer review history

# Peer review: Reviewer response for version 2

**DOI:** 10.21956/wellcomeopenres.18998.r46238

**Published:** 2021-10-25

**Authors:** Victoria Simms

**Affiliations:** 1 School of Psychology, Ulster University, Coleraine, UK

All comments have been sufficiently addressed- thank you!

Is the work clearly and accurately presented and does it cite the current literature?

Yes

If applicable, is the statistical analysis and its interpretation appropriate?

Yes

Are all the source data underlying the results available to ensure full reproducibility?

Yes

Is the study design appropriate and is the work technically sound?

Yes

Are the conclusions drawn adequately supported by the results?

Yes

Are sufficient details of methods and analysis provided to allow replication by others?

Yes

Reviewer Expertise:

Developmental psychology

I confirm that I have read this submission and believe that I have an appropriate level of expertise to confirm that it is of an acceptable scientific standard.

# Peer review: Reviewer response for version 1

**DOI:** 10.21956/wellcomeopenres.18601.r44457

**Published:** 2021-07-07

**Authors:** Paige Terrien Church

**Affiliations:** 1 Department of Newborn and Developmental Paediatrics, Sunnybrook Health Sciences Centre, Toronto, ON, Canada

This article evaluates through a survey as well as qualitative data parental preferences and priorities for research and communication of the findings. The survey was complex with differing formats including rating scales, multiple choice ranking and open ended questions. The open ended questions were reviewed for thematic content. What I think is particularly important (although I object to the wording of 'problem' and would favor a designation of 'challenge' score) is this concept of conceptualizing the replies in the context of the parental experience. 

 The results are important as they represent needed information on what is important to parents including a focus on positive outcomes rather than a deficit based focus. They also looked for a focus on protective upstream strategies rather than downstream findings. Parents identified that which has been identified in the literature, that there is a knowledge gap in the real world amongst educators and health care workers. The findings did not seem to be affected by the status of the challenges experienced.

Is the work clearly and accurately presented and does it cite the current literature?

Yes

If applicable, is the statistical analysis and its interpretation appropriate?

Yes

Are all the source data underlying the results available to ensure full reproducibility?

Partly

Is the study design appropriate and is the work technically sound?

Yes

Are the conclusions drawn adequately supported by the results?

Yes

Are sufficient details of methods and analysis provided to allow replication by others?

Yes

Reviewer Expertise:

My area of research is in neonatal outcomes.

I confirm that I have read this submission and believe that I have an appropriate level of expertise to confirm that it is of an acceptable scientific standard.

# Response

**Published:** 2021-09-01

**Authors:** Lorna Ginnell

**Affiliations:** University of Edinburgh, UK

Thank you very much for your thoughtful comments. We agree that “challenge” rather than “problem” is more appropriate and have amended this throughout the text and figures.

# Peer review: Reviewer response for version 1

**DOI:** 10.21956/wellcomeopenres.18601.r44531

**Published:** 2021-07-07

**Authors:** Victoria Simms

**Affiliations:** 1 School of Psychology, Ulster University, Coleraine, UK

This paper explores parent priorities for research and information dissemination in relation to preterm birth and its consequences. This type of stakeholder engagement is warmly welcomed. 148 parents completed the survey from across the UK. Parents prioritised research that will help identify protective factors for positive childhood outcomes. Parents also want to receive information from within the first year of their child's life. Training professionals who work with children born preterm, along with intervention research should be prioritised. The point made around strengthening direct pathways between research and clinical services is an excellent suggestion and the potential pitfalls are also outlined. 

 Some specific points to be addressed:

1. The literature review sets up the paper for the reader to expect a replication of a research priority setting like that of Zeitlin et al. (2020) ^1^ - this was addressed in the current study, but only through one question. Perhaps a little reworking of the introduction to increase the emphasis on the impact of attitudes on participation in studies and the novelty around the questions asked to parents about when they would like to receive information would be helpful.  
2. Were the questions about whether children experienced challenges in different domains taken from a previous publication? If no, how were the domains identified and justified?
3. What was the geographical spread of the participants? Were there any geographical differences - is the sample representative of the UK population?
4. In the questions displayed in Figure - what do the response values represent? I think clarity is needed here in the text. From the Figure explanation I think parents ask to rank each option contained within each question.
5. In Figure 2 is there any data on what parents were suggesting under "other" in response to QA?
6. Figure 3 is counterintuitively presented - would it not be better to have "not at all" bars on the left hand side of the graph, etc. Figure 4 and 5 would benefit from %s being added too. 
7. I think the sample size should be discussed as a limitation in the discussion section. 

Is the work clearly and accurately presented and does it cite the current literature?

Yes

If applicable, is the statistical analysis and its interpretation appropriate?

Yes

Are all the source data underlying the results available to ensure full reproducibility?

Yes

Is the study design appropriate and is the work technically sound?

Yes

Are the conclusions drawn adequately supported by the results?

Yes

Are sufficient details of methods and analysis provided to allow replication by others?

Yes

Reviewer Expertise:

Developmental psychology

I confirm that I have read this submission and believe that I have an appropriate level of expertise to confirm that it is of an acceptable scientific standard, however I have significant reservations, as outlined above.

## References

[1] : Priorities for collaborative research using very preterm birth cohorts. *Arch Dis Child Fetal Neonatal Ed* .2020;105(5) : 10.1136/archdischild-2019-317991 538-544 10.1136/archdischild-2019-317991 32029530PMC7547907

# Response

**Published:** 2021-09-01

**Authors:** Lorna Ginnell

**Affiliations:** University of Edinburgh, UK

Thank you very much for this insightful review. We have responded to each comment in turn and detailed any changes the have been made.

1. Thank you for this very helpful suggestion. We have amended the introduction as suggested, to provide what we hope is a more focused and informative background to the current study. We have removed a few sentences focusing on previous research about priority setting for neonatal outcomes, and added several sentences highlighting the importance of understanding attitudes towards research and communication preferences in more general terms. The goal is to make it more clear that existing priority-setting work motivates our study because it reveals the differences between parent and clinical / academic perspectives.  This provides an important part of the justification for this new work.
2. No, these questions were developed for the study, drawing on domains relevant to our ongoing longitudinal cohort study as well as more informal sources.  A description of this is provided in paragraph one of the "Materials" section. In recognition of the implicit limitation noted by the reviewer here, we have also added some new text in the discussion to acknowledge this limitation.
3.  We have added information on geographical spread of the participants to the methods section (56 England, 49 Scotland, 4 Wales, 1 Northern Ireland, 38 UK country not specified), and have noted the uneven geographical split as a limitation in the discussion section.
4. We have augmented the explanation of how participants were asked to respond to questions displayed in Figure 1 within the figure legend and within the methods section of the text. We hope we have interpreted this request correctly.
5. Unfortunately, we do not have data on this. “Other” was ranked as the least preferable method of receiving information by 70% of participants and only 2.7% ranked it as the most preferable method of receiving information, so we are hopeful we have not lost too much information here, though we appreciate we have missed the opportunity to represent the ideas of that 2.7%.  
6.  Figure 3 has been edited as suggested and percentages have been added to figures 4 and 5.
7. We have added a note on this to the limitations section.
